# Association between cigarette smoking status and voting intentions: Cross sectional surveys in England 2015-2020

**DOI:** 10.1186/s12889-021-12304-4

**Published:** 2021-12-11

**Authors:** Sharon Cox, Jamie Brown, Cheryl McQuire, Frank de Vocht, Emma Beard, Robert West, Lion Shahab

**Affiliations:** 1 Department of Behavioural Science and Health, University College London, 1-19 Torrington Place, London, UK; 2 Population Health Sciences Institute, University of Bristol, Bristol, UK; 3 NIHR School for Public Health Research, Bristol, UK

**Keywords:** Tobacco, Health inequality, Inequity, Political participation, Health behaviour, Voting, Smoking

## Abstract

**Background and aims:**

Cigarette smoking takes place within a cultural and social context. Political views and practices are an important part of that context. To gain a better understanding of smoking, it may be helpful to understand its association with voting patterns as an expression of the political views and practices of the population who smoke. This study aimed to assess the association between cigarette smoking and voting intentions and to examine how far any association can be explained by sociodemographic factors and alcohol use.

**Methods:**

Pooled monthly representative repeat cross-sectional household surveys of adults (16+) in England (*N* = 55,482) between 2015 and 2020 were used to assess the association between cigarette smoking status and voting intentions, and whether this was accounted for by age, occupational grade, gender, region and alcohol use. Voting intention was measured by asking ‘How would you vote if there were a General Election tomorrow?’ Respondents chose from a list of the major English political parties or indicated their intention not to vote.

**Results:**

In adjusted multinomial regression, compared with intending to vote Conservative (majority party of government during the period), being undecided (aOR1.22 [1.13-1.33] <0.001), intending to vote Labour (aOR1.27 [1.16-1.36] <0.001), to vote “Other” (aOR1.54 [1.37-1.72] <0.001), or not to vote (aOR1.93 [1.77-2.11] <0.001) was associated with higher odds of current relative to never smoking rates. Intending to vote for the Liberal Democrats was associated with a significant lower odds of current smoking prevalence (aOR0.80 [0.70-0.91] <0.001) compared with intending to vote Conservative.

**Conclusions:**

Controlling for a range of other factors, current as compared with never-smokers appear more likely to intend not to vote, to be undecided, to vote for Labour or a non-mainstream party, and less likely to vote for the Liberal Democrats, compared with the Conservative party.

**Supplementary Information:**

The online version contains supplementary material available at 10.1186/s12889-021-12304-4.

## Introduction

Poor general health, wealth inequality and higher mortality are all negatively associated with voter turnout [1–3]. Of a range of health behaviours, evidence shows cigarette smoking status is strongly negatively correlated with poor voter turnout [4], although the reasons why are not well established. It is unlikely that smoking directly influences voting, but instead because of a shared association with social disadvantage, it is a wider set of health, social and cultural factors that influences political beliefs and participation [3, 5]. However, because smoking is also more prevalent within poorer communities, by not voting, it is possible smokers from the most disadvantaged communities are underrepresented in elections. This is a disadvantage because communities with higher numbers of smokers are particularly affected by the spending priorities of political parties since these determine resources for tobacco control activities. Given the substantial health burden and inequalities caused by smoking [6], and the pivotal role that political parties have in smoking prevention measures and health and community spending, it is important to understand how sociodemographic characteristics, smoking behaviours and voting intentions are linked. The purpose of this study is to examine the association of smoking status, and sociodemographic characteristics, with voting intentions in England between 2015 and 2020.

Tobacco smoking is a community health issue, disproportionately affecting individuals and families of lower economic status by reducing life expectancy and increasing morbidity; general health is poorer among life-time smokers than never-smokers [7]. Tobacco retail outlet density is positively correlated with neighbourhood deprivation [8], at the same time reductions to smoking cessation services have been highest in the most deprived areas of England [9]. While there has been a reduction in smoking prevalence across the socioeconomic gradient, substantial tobacco related health inequalities remain [10–13]. In England, tobacco smoking rates are currently twice as high in those in routine and manual occupations (20.8%) compared with managerial and professional occupations (9.9%) [14]; prevalence rates are even higher amongst those with competing health and social needs (e.g., experiencing homelessness or insecure housing, food poverty) [7, 15]. Taken together, this underlines that tobacco smoking continues to be a large contributor to health and social inequalities. Political parties committed to improving community health should be focusing on the burdens caused by tobacco smoking, especially in view of the latest promises by the UK Government to ‘level up’ the opportunities for those living in deprived towns through increased social participation and mobility [16].

Previous research from Britain and the Republic of Ireland has shown that a greater proportion of smokers do not vote [1, 3, 4]. One study in Britain measuring the relationship between voting behaviour and indicators of health showed smokers were consistently less likely to turnout to vote across the 1979, 1987 and 1997 general elections [4]. When people who smoke do vote, their political party affiliation has not been well documented. In the few published examples examining poor health, and voting more broadly, patterns of voting are mixed. For example, in the 2017 French presidency elections, poorer health status was positively correlated with voting for far-right candidate Marie Le-Pen compared with voting for centrist candidate Emmanuel Macron, the current president [17]. In the 1997 general election in the Republic of Ireland, however, indicators of deprivation, self-reported poorer quality of life health status, and smoking were correlated with left-wing voting even within a traditionally right-leaning ruling country [1]. In Sweden, one study showed higher political mistrust among daily smokers, with those reporting high distrust or no opinion on government also being less likely to report trying to quit [18]. While in England there have been no studies directly examining trust in political parties or systems among smokers, there have been media stories and reports of the tobacco industry providing funding to politicians across a range of parties to gain influence (e.g., [19]). Any deterioration of trust in the political system may be especially heightened among certain groups of smokers. There is some evidence of scepticism as to whether political parties truly care about them and their health, as they perceive the revenue raised through their tobacco purchasing as beneficial for the government [20]. This trust is also largely believed to be influenced by the media, including newspapers, who some suggest have the power to undermine the credibility of politicians [21].

In the same way there is a shared association between health, social disadvantage and voting, there may also be with newspaper readership. Social capital and status, income and health status are also known to be important predictors of newspaper readership [22–24], and newspaper readership is influential in voting – both through persuading readers and, in the UK, having standing affiliations with political parties during elections. While there is evidence on the relationship between health behaviours and newspaper readership from outside the UK, there is to date very little from within. To expand the literature in this field, we therefore also consider newspaper readership in a sensitivity analyses here.

For those facing health and social difficulties voting may not be a priority, and, when it is, a wider set of cultural factors may be perceived as more important to improving one’s life than health. For some smokers, quitting smoking and improving one’s health may be perceived as having little impact on one’s lived circumstances. The relative health burden and inequalities caused by smoking and its related circumstances could lead to a type of ‘political exclusion’ inequality, making certain groups within the population uncountable and underrepresented [3].

Here we explore the association between smoking status, associated demographic (sex, age, social grade, region) and health characteristics (alcohol use) and voting intentions in England between 2015 and 2020. We also a measure of alcohol use as there is a strong association between smoking and alcohol consumption and alcohol use undermines quitting [25–27], thus alcohol may serve as another proxy health measure for voting intentions. These intentions were not bound to any specific election (i.e., 8th June 2017, 12th December 2019), instead the question was theoretical, asking “how would you vote if there was an election tomorrow?” UK Newspapers are widely perceived as right- or left-leaning, with newspaper readership reflecting broad political orientation. As voting intentions are sensitive to changes, depending on particular issues and leaders of political parties, we therefore include a sensitivity analysis here replacing voting intention with newspaper readership.

Using monthly cross-sectional data from the Smoking Toolkit Study, this study aimed to examine the association between smoking status and voting intentions. Specifically, we aimed to investigate two research questions:

1. Is there an association between smoking status (smoker, former-smoker, never smoker) and party voting intentions?

2: Is any association between voting intentions and smoking status independent of sociodemographic characteristics (sex, age, socioeconomic status as measured by occupational grade, region) and alcohol use?

## Methods

### Design and setting

 Data were collected between February 2015 and February 2020 from the ongoing Smoking Toolkit Study (STS), a monthly repeated cross-sectional survey of a representative sample of adults in England [28]. The STS uses a hybrid of random location and quota sampling to select a new sample of approximately 1,700 adults (aged ≥16 years) each month. Locations are randomly selected from around 170,000 output areas in England, stratified by a geodemographic classification of the population. Interviews are performed with one household member until quotas based on factors influencing the probability of being at home (e.g. sex, age, working status) are fulfilled. Comparisons with sales data and other national surveys show that the STS recruits a representative sample of the population in England with regards to key demographic variables, smoking prevalence, and cigarette consumption [19].

### Ethical approval and consent to participate

Ethical approval for the STS was granted by the UCL Ethics Committee (ID 0498/001). All participants are treated in accordance with the principles of the Declaration of Helsinki. Written informed consent is obtained by all participants. The data is not collected by UCL and is anonymised when received by the research team.

### Measures

#### Outcome variable

 In order to measure party voting intentions, respondents were asked “How would you vote if there were a General Election tomorrow?” (1) Conservative (reference category; traditionally a right leaning party), (2) Labour (traditionally a left leaning party), (3) Liberal Democrat (considered a centrist party), (4) Green Party (traditionally a left leaning party), (5) UK Independence Party (right leaning party), (6) Other, (7) intended not to vote, (8) Undecided, (9) Refused.

#### Explanatory variable

 Smoking status was determined by asking, “Which of the following best applies to you” (1) I smoke cigarettes (including hand-rolled) every day, (2) I smoke cigarettes (including hand-rolled), but not every day, (3) I do not smoke cigarettes at all, but I do smoke tobacco of some kind (e.g. pipe, cigar or shisha), (4) I have stopped smoking completely in the last year, (5) I stopped smoking completely more than a year ago, (6) I have never been a smoker (i.e. smoked for a year or more). Those who reported currently smoking cigarettes or tobacco of another type were considered to be a smoker. All of those who reported having stopped smoking within the last year or before were considered former smokers. All others were considered never-smokers.

### Covariates

Sex was categorised by female/other, and age by category (16-24, 25-34, 35-44, 45-54, 55-64, and ≥65 years).

#### Occupational social grade

 As measured by the National Readership Survey [29], comprises AB (higher and intermediate managerial, administrative and professional), C1 (supervisory, clerical and junior managerial, administrative and professional), C2 (skilled manual workers), D (semi-skilled and unskilled manual workers) and E (state pensioners, casual and lowest-grade workers, unemployed with recourse to state benefits.

#### Region

 Region is presented by four divisions of England, North, Central, South and London.

#### Alcohol

 The AUDIT [30] score was used as a continuous measure for alcohol drinking and associated behaviour and is a known confounder of associations between smoking and health outcomes. The AUDIT is a widely used measure of alcohol use designed to indicate alcohol use which is potentially harmful to health. A score between 0 and 7 indicate the lower risk category, scores of 8-15 indicate increasing risk, 16-19 higher risk and those of 20+ indicate possible dependence.

#### Newspaper readership

 Participants were asked which daily national newspaper they read regularly (e.g., Daily Mail, The Sun, The Telegraph, The Guardian, (list not exhaustive)).

### Analyses

This study was preregistered on the Open Science Framework: https://osf.io/fq7rd/. Within the protocol current smoker was indicated as the reference category; however, because never smokers are the largest category this forms the reference category here instead. Analysis was conducted using SPSS v. 26. Data analysis was conducted on complete cases for all variables (<5% missing at random) and descriptive data were weighted to match the English population profile on age, social grade, region, tenure, ethnicity, and working status within sex. The dimensions are derived monthly from a combination of the English 2011 census, Office for National Statistics mid-year estimates, and an annual random probability survey conducted for the National Readership Survey.

### Causal pathways

The covariate adjustment set was determined by constructing a directed acyclic graph (DAG) (Supplementary Fig. 1). Based on the published literature, the DAG illustrates the hypothesized causal and mediated pathways between smoking and observed health and social factors as well as unobserved latent factors, not able to be captured here, and their relationship to voting.

For research question 1, we used a multinomial regression model to estimate the unadjusted association (presenting the 95% confidence interval [CI]) between smoking status (never smoker as reference category) and voting intentions on unweighted data. The Conservative party was selected as the reference category because it was the government party at the time of data collection. For research question 2, we used a multinomial model to estimate the associations adjusting for AUDIT scores (as a continuous variable) and sociodemographic characteristics (categorical variables) on unweighted data. Across all models presented below, Goodness-of-fit tests indicated the full model statistically significantly predicted the dependent variable better than the intercept-only model alone (Likelihood ratio < 0.001). Independence of observations and multicollinearity were evaluated with simple correlations among the independent variables.

Sensitivity analysis: To assess the extent to which associations with voting intention reflect associations with political orientation, we planned to repeat the models with newspaper readership replacing party voting intention. However, from a visual inspection of the frequency with which people reported newspaper readership, the individual categories of newspapers did not correspond with voting intention. As there is a larger number and readership of right-leaning and mixed papers, we decided that because some papers have historically switched their leaning and to reduce skewness of right leaning, only two papers of each leaning, based on their clear political affiliation [31] would be selected for analysis. Guardian and Mirror were coded as left-leaning, Independent and Metro coded as centrist, and the Daily Express and Daily Mail as right-leaning, with mixed readership indicating readership across leanings.

## Results

### Participant characteristics

55,482 (complete cases) participants responded between 2015 and 2020 (51% female *n*=28,303; mean age 47.37 years (*sd* = 1.7)). Table 1 provides the weighted descriptive statistics by party voting intention. Respondents most commonly reported intending to vote Labour (27.1%), with 19.8% Conservative, and a further 6.8% Liberal Democrats, although almost a quarter reported that they were undecided, with the remainder refusing to answer, not planning to vote or selecting an ‘Other’ party. In a follow-up question, responses indicated Green Party 2.6%, UK Independence Party 1.8%, other 0.7%. and British National Party 0.5%.

**Table 1:** Descriptive data of participants by party voting intentions

	Total	Conservative	Labour	Liberal Democrats	Other	Would not vote	Undecided	Refused
**% (n)**	100 (55,482)	19.8 (11,006)	27.1 (15,013)	6.2 (3461)	5.9 (3266)	12.2 (6767)	24.5 (13,595)	4.3 (2375)
**Smoking status % (n)**								
Never smoker	65.4 (36,237)	65.8 (7349)	65.7 (10,144)	69.8 (2358)	57.3(1832)	58.7 (3841)	67.1 (8957)	73.5 (1756)
Former-smoker	18.2 (9964)	22.5 (2515)	16.5 (2539)	20.2 (682)	23.2 (741)	13.8 (904)	16.7 (2236)	14.5 (347)
Current smoker	16.4 (9253)	11.7 (1302)	17.8 (2748)	10 (338)	19.5 (625)	27.4 (1793)	16.2 (2162)	11.9 (285)
**Newspaper readership % (n)**								
Daily Mail	32.7 (3640)	62 (1612)	13.9 (523)	17.5 (128)	32.3 (229)	32.2 (238)	33.8 (739)	37.5 (171)
Daily Express	7.6 (801)	12.5 (326)	3.1 (116)	2.5 (18)	11.8 (84)	7.8 (58)	7.4 (162)	8.1 (37)
The Guardian	27.1 (2946)	7 (183)	38.7 (1454)	54.5 (398)	31.2 (221)	17.2 (127)	21.8 (476)	19.1 (87)
Daily Mirror	10.6 (1341)	6.3 (164)	16.8 (632)	5.3 (39)	6.7 (48)	15.5 (115)	13.4 (292)	11.2 (51)
The Independent	1.2 (153)	1.1 (27)	1.7 (64)	1.2 (9)	2.3 (16)	0.9 (7)	1.3 (29)	0.2 (1)
Metro	20.6 (2299)	11.1 (290)	25.8 (968)	19 (139)	15.7 (111)	26.4 (195)	22.3 (487)	23.9 (109)
**Age**								
16-24	13.6 (8194)	6.5 (729)	19.4 (2996)	9.9 (335)	11.6 (371)	21.3 (1392)	15.8 (2117)	10.6 (254)
25-34	13.5 (8059)	7.3 (814)	17.4 (2690)	10.2 (346)	11.5 (368)	21.4 (1398)	16.3 (2184)	10.8 (259)
35-44	14.1 (7782)	9.4 (1052)	15.7 (2427)	15.5 (523)	13.4 (428)	16.2 (1061)	14.7 (1960)	13.8 (331)
45-54	12.6 (8049)	13.7 (1532)	14.4 (2225)	16.8 (568)	16.7 (534)	13.8 (900)	14.4 (1927)	15.2 (363)
55-64	16.2 (8584)	18.1 (2022)	13.8 (2132)	18.2 (616)	19.1 (611)	12.1 (789)	14.9 (1995)	17.5 (419)
65+	27.5 (14,817)	45 (5021)	19.2 (2970)	29.3 (991)	27.7 (886)	15.3 (1004)	23.8 (3179)	32 (766)
**Sex**								
Male	51.2 (27,875)	54.9 (6129)	50.2 (7757)	51.6 (1745)	56.6 (1809)	50.6 (3309)	44.4 (5930)	50 (1196)
Female	48.8 (27,610)	45.1 (5041)	49.8 (7683)	48.4 (1634)	43.4 (1389)	49.4 (3235)	55.6 (7432)	50 (1196)
**Occupation social grade**								
AB	26.5 (13,140)	31.4 (3510)	23.2 (3581)	43.6 (1474)	25.1 (804)	13 (849)	23.2 (3099)	26 (623)
C1	34.2 (19,150)	35.9 (4006)	34.6 (5343)	36 (1217)	34 (1086)	28.9 (1889)	35.8 (4788)	34.3 (821)
C2	18.6 (10,535)	17.9 (2005)	18.1 (2795)	10.6 (357)	21.2 (677)	24.7 (1617)	19.8 (2646)	18.3 (438)
D	11.6 (6563)	8.7 (973)	13 (2010)	5.1 (171)	10.2 (326)	19.7 (1290)	12.5 (1673)	11.7 (280)
E	9.1 (5037)	6.1 (676)	11.1 (1711)	4.7 (160)	9.5 (205)	13.7 (899)	8.7 (1156)	9.6 (230)
**Region**								
South of England	23.9 (12,606)	29.6 (3306)	15.4 (2383)	33.3 (1125)	26.4 (845)	18.3 (1199)	24.4 (3261)	20.4 (487)
London	17.4 (9601)	10.4 (1163)	24.6 (3799)	19.8 (669)	13.7 (439)	15.6 (1018)	14.7 (1969)	22.7 (544)
Central England	30.8 (16,782)	33.6 (3747)	25.1 (3874)	26 (878)	28.8 (919)	35.6 (2329)	31.4 (4189)	35.4 (846)
North of England	23.9 (16,479)	26.4 (2947)	34.9 (5383)	20.9 (707)	31.1 (993)	30.5 (1996)	29.5 (3938)	21.5 (515)
**AUDIT Score** mean score (SD)	3.31 (3.72)	3.36 (3.64)	3.41 (4.03)	4.00 (3.767)	4.00 (4.05)	2.97 (4.02)	3.08 (3.57)	2.39 (3.07)

Data is weighted so does not equally match total across all variables

**Fig. 1:**
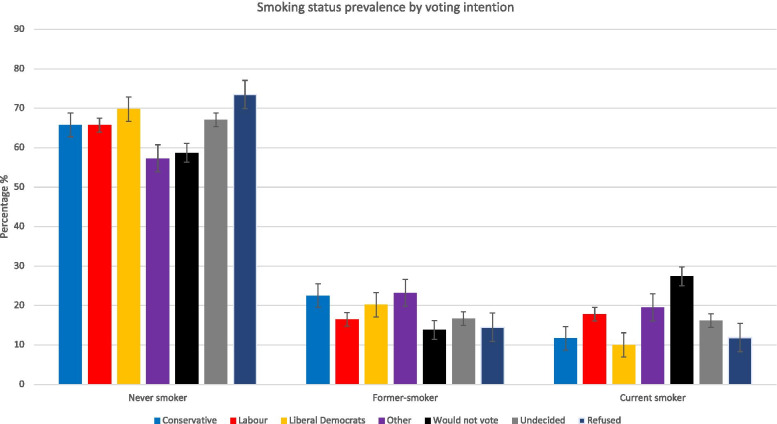
Smoking prevalence (never smoker, former-smoker and current smoker) by voting intention. Respondents were asked “How would you vote if there were a General Election tomorrow?” Bar represent the 95% population proportion confidence interval

Prevalence of smoking status across the whole sample was, 65.4% (95% CI 65.43-66.12) never-smoker, 17.3% (95% CI 17.11 – 17.61) former-smoker, and 16.9% (95% CI 16.6-17.2) current smoker. Figure 1 presents smoking status prevalence by voting intentions; those intending not to vote represented the highest percentage of current smokers 27.4% (95% CI 26.4-28.5) and the lowest of former smokers 13.8% (95% CI 13.01-14.7%). Conversely, those who refused to answer had the highest percentage of never smokers (73.5%; 95% CI 72.13-75.66), followed by those intending to vote Liberal Democrat 69.8% (95% CI 68.22-71.32) who also represented the fewest current smokers 10% (95% CI 9.09-11.16%). Those intending to vote Liberal Democrat and ‘Other’ had the highest AUDIT scores, although the mean score for all groups was within the lower risk drinking category (Table 1).

### RQ1: Voting intention

In unadjusted analysis, compared with intending to vote Conservative, being undecided (OR 1.38 95% CI[1.25 – 1.45] *p*<0.001), intending to vote Labour (OR 1.48 [1.38-1.52] *p*<0.001), “Other” (OR 1.80 [1.61-2.00] *p*<0.001) or intending not to vote (OR 2.57 [2.38-2.79] *p*<0.001) was associated with higher odds to be a current - relative to never - smoker, while intending to vote Liberal Democrat (OR 0.78 [0.69-0.81] *p*<0.001) or refusal to answer (OR 0.59 [0.52-0.67] *p*<0.001) was associated with lower odds to be a current smoker (Table 2).

**Table 2:** Multinomial regression unadjusted and full adjusted model; voting intentions, sociodemographic characteristics and alcohol use

	Labour		Lib Dems		Other		Would not vote		Undecided		Refused	
	OR (95% CI) *p* value	Adj OR (95% CI) *p* value	OR (95% CI) *p* value	Adj OR (95% CI) *p* value	OR (95% CI) *p* value	Adj OR (95% CI) *p* value	OR (95% CI) *p* value	Adj OR (95% CI) *p* value	OR (95% CI) *p* value	Adj OR (95% CI) *p* value	OR (95% CI) *p* value	Adj OR (95% CI) *p* value
**Smoking status**												
Never smoker	1.00	1.00	1.00	1.00	1.00	1.00	1.00	1.00	1.00	1.00	1.000	1.00
Former smoker	0.76 (0.71-0.81) **<0.001**	1.10 (1.03-1.18) **0.006**	0.88 (0.80-0.96) **0.007**	0.99 (0.89-1.09) 0.79	1.17 (1.06-1.29) **0.002**	1.34 (1.21-1.48) **<0.001**	0.71 (0.65 – 0.78) **<0.001**	1.13 (1.03-1.24) **0.007**	0.73 (0.69-0.79) **<0.001**	1.03 (0.96-1.10) 0.41	0.59 (0.52-0.67) **<0.001**	0.80 (0.70-0.91) **0.001**
Current smoker	1.48 (1.38-1.52) **<0.001**	1.27 (1.16-1.36) **<0.001**	0.78 (0.69-0.81) **<0.001**	0.80 (0.70-0.91) **0.001**	1.80 (1.61-2.00) **<0.001**	1.54 (1.37-1.72) **<0.001**	2.57 (2.38-2.79) **<0.001**	1.93 (1.77-2.11) **<0.001**	1.38 (1.25 – 1.45) **<0.001**	1.22 (1.13-1.33) **<0.001**	0.93 (0.81-1.07) 0.32	0.97 (0.84-1.11) 0.64
**Age**												
65 +	1.00	1.00	1.00	1.00	1.00	1.00	1.00	1.00	1.00	1.00	1.00	1.00
55-64	1.75 (1.61-1.90) **<0.001**	1.87 (1.67-1.98) **<0.001**	1.54 (1.36-1.73) **<0.001**	1.51 (1.34-1.71) **<0.001**	1.77 (1.56-2.00) **<0.001**	1.79 (1.59-2.03) **<0.001**	1.95 (1.74-2.18) **<0.001**	2.09 (1.86-2.35) **<0.001**	1.54 (1.42-1.66) **<0.001**	1.65 (1.52-1.80) **<0.001**	1.34 (1.17-1.54) **<0.001**	1.45 (1.26-1.66) **<0.001**
45-54	2.42 (2.24-2.62) **<0.001**	2.54 (2.35-2.76) **<0.001**	1.92 (1.71-2.15) **<0.001**	1.82 (1.62-2.05) **<0.001**	2.02 (1.79-2.27) **<0.001**	2.07 (1.83-2.34) **<0.001**	2.79 (2.50-3.10) **<0.001**	3.15 (2.82-3.52) **<0.001**	1.91 (1.77-2.07) **<0.001**	2.10 (1.93-2.28) **<0.001**	1.50 (1.31-1.71) **<0.001**	1.61 (1.41-1.85) **<0.001**
35-44	3.76 (3.45-4.08) **<0.001**	3.76 (3.45-4.10) **<0.001**	2.41 (2.13-2.72) **<0.001**	2.34 (1.98-2.54) **<0.001**	2.34 (2.04-2.68) **<0.001**	2.40 (2.11-2.75) **<0.001**	4.83 (4.33-5.39) **<0.001**	5.34 (4.77-5.98) **<0.001**	2.74 (2.52-2.98) **<0.001**	2.97 (2.72-3.24) **<0.001**	1.95 (1.69-2.25) **<0.001**	1.94 (1.67-2.25) **<0.001**
25-34	5.28 (4.81-5.73) **<0.001**	5.02 (4.59-5.50) **<0.001**	2.05 (1.79-2.35) **<0.001**	2.03 (1.96-0.271) **<0.001**	2.55 (2.23-2.92) **<0.001**	2.53 (2.20-2.91) **<0.001**	8.34 (7.49-9.29) **<0.001**	8.28 (7.39-9.27) **<0.001**	3.94 (3.61-4.30) **<0.001**	4.10 (3.75-4.49) **<0.001**	2.02 (1.74-2.35) **<0.001**	1.96 (1.67-2.29) **<0.001**
16-24	6.89 (6.25-7.59) **<0.001**	7.10 (6.40-7.83) **<0.001**	2.10 (1.80-2.45) **<0.001**	2.30 (1.96-2.71) **<0.001**	2.90 (2.50-3.37) **<0.001**	2.99 (2.55-3.50) **<0.001**	9.74 (8.65-10.95) **<0.001**	10.36 (9.15-11.74) **<0.001**	4.67 (4.23-5.15) **<0.001**	5.19 (4.68-5.75) **<0.001**	2.23 (1.88-2.64) **<0.001**	2.44 (2.05-2.91) **<0.001**
**Sex**												
Female	1.00	1.00	1.00	1.00	1.00	1.00	1.00	1.00	1.00	1.00	1.00	1.00
Male	0.89 (0.84-0.93) **<0.001**	0.88 (0.84-0.93) **<0.001**	0.90 (0.84-0.98) **0.009**	0.87 (0.80-0.94) **<0.001**	1.14 (1.05-1.23) **0.001**	1.09 (1.01-1.18) **0.04**	0.94 (0.88-0.99) **0.03**	0.94 (0.88-1.00) 0.54	0.70 (0.67-0.74) **<0.001**	0.72 (0.68-0.76) **<0.001**	0.85 (0.78-0.93) **<0.001**	0.72 (0.68-0.76) **<0.001**
**Occupation social grade**												
AB	1.00	1.00	1.00	1.00	1.00	1.00	1.00	1.00	1.00	1.00	1.00	1.00
C1	1.27 (1.19-1.36) **<0.001**	1.01 (0.95-1.08) 0.73	0.70 (0.64-0.77) **<0.001**	0.69 (0.63-0.76) **<0.001**	1.18 (1.07-1.31) **0.002**	1.10 (0.99-1.22) 0.07	1.88 (1.71-2.06) **<0.001**	1.43 (1.30-1.58) **<0.001**	1.33 (1.25-1.43) **<0.001**	1.10 (1.03-1.18) **0.007**	1.16 (1.03-1.30) **0.02**	1.00 (0.88-1.12) 0.95
C2	1.29 (1.20-1.39) **<0.001**	1.13 (1.04 – 1.22) **0.002**	0.41 (0.37-0.47) **<0.001**	0.43 (0.38-0.49) **<0.001**	1.45 (1.29-1.61) **<0.001**	1.36 (1.22-1.52) **<0.001**	3.28 (2.98-3.60) **<0.001**	2.63 (2.38-2.91) **<0.001**	1.43 (1.33-1.54) **<0.001**	1.27 (1.18-1.37) **<0.001**	1.23 (1.08-1.40) **0.002**	1.08 (0.95-1.24) 0.23
D	1.84 (1.69-2.00) **<0.001**	1.52 (1.39-1.66) **<0.001**	0.43 (0.38-0.50) **<0.001**	0.46 (0.39-0.54) **<0.001**	1.36 (1.18-1.56) **<0.001**	1.29 (1.12-1.48) **<0.001**	5.33 (4.81-5.91) **<0.001**	3.95 (3.54-4.41) **<0.001**	1.88 (1.73-2.04) **<0.001**	1.58 (1.44-1.72) **<0.001**	1.63 (1.41-1.89) **<0.001**	1.33 (1.15-1.55) **<0.001**
E	2.09 (1.89-2.31) **<0.001**	1.98 (1.78 – 2.20) **<0.001**	0.54 (0.45-0.64) **<0.001**	0.65 (0.54-0.77) **<0.001**	1.64 (1.40-1.91) **<0.001**	1.74 (1.47-2.04) **<0.001**	4.69 (4.15-5.30) **<0.001**	4.23 (3.71-4.82) **<0.001**	1.73 (1.56-1.92) **<0.001**	1.65 (1.48-1.84) **<0.001**	1.78 (1.50-2.11) **<0.001**	1.53 (1.28-1.83) **<0.001**
**Region**												
North	1.00	1.00	1.00	1.00	1.00	1.00	1.00	1.00	1.00	1.00	1.00	1.00
Central	0.55 (0.51-0.58) **<0.001**	0.55 (0.51-0.59) **<0.001**	0.96 (0.86-1.08) **<0.001**	0.97 (0.86-1.09) 0.60	0.71 (0.64-0.78) **<0.001**	0.73 (0.66-0.82) **<0.001**	0.93 (0.86-1.01) 0.07	0.92 (0.85-1.00) 0.05	0.84 (0.78-0.89) **<0.001**	0.82 (0.76-0.88) **<0.001**	1.29 (1.14-1.46) **<0.001**	1.22 (1.07-1.38) **0.002**
South	0.41 (0.38-0.43) **<0.001**	0.47 (0.43-0.50) **<0.001**	1.50 (1.34-1.66) **<0.001**	1.48 (1.33-1.65) **<0.001**	0.82 (0.74-0.91) **<0.001**	0.90 (0.82-1.00) 0.05	0.55 (0.51-0.60) **<0.001**	0.68 (0.62-0.75) **<0.001**	0.73 (0.69-0.79) **<0.001**	0.81 (0.76-0.87) **<0.001**	0.86 (0.76-0.98) **0.03**	0.90 (0.78-1.02) 0.10
London	1.78 (1.61-1.90) **<0.001**	1.55 (1.42-1.69) **<0.001**	2.62 (2.30-2.99) **<0.001**	2.44 (2.14-2.79) **<0.001**	1.11(0.96-1.27) **<0.001**	1.08 (0.94-1.24) 0.28	1.27 (1.15-1.42) **<0.001**	1.09 (0.97-1.22) 0.14	1.26 (1.15-1.38) **<0.001**	1.11 (1.01-1.22) **0.04**	2.83 (2.44-3.28) **<0.001**	2.43 (2.10-2.82) **<0.001**
**AUDIT Score**												
	0.98 (0.97-0.99) **<0.001**	0.96 (0.96-0.97) **<0.001**	1.02 (1.01-1.03) **<0.001**	1.01 (1.00-1.02) 0.11	1.02 (1.01-1.03) **<0.001**	1.00 (0.99-1.01) 0.33	0.95 (0.94-0.96) **<0.001**	0.94 (0.93-0.94) **<0.001**	0.96 (0.95-0.97) **<0.001**	0.95 (0.94-0.96) **<0.001**	0.90 (0.88-0.91) **<0.001**	0.90 (0.88-0.91) **<0.001**

Would vote Conservative is the reference category. Bold indicates statistical significance

Compared with intending to vote Conservative, being undecided (OR0.73 [0.69-0.79] *p*<0.001), intending to vote Labour (OR 0.76 [0.71-0.81] *p*<0.001), Liberal Democrat (OR 0.88 [0.80-0.96] p=0.007), intending not vote (OR 0.71 [0.65 – 0.78] *p*<0.001) or refusal to answer (OR 0.59 [0.52-0.67] *p*<0.001) was associated with a lower odds of former relative to never smoking, while ‘Other’ voting intention was associated with higher odds of former smoking (OR 1.17 (1.06-1.29) *p=*0.002).

### RQ2: Voting intention (fully adjusted model)

In fully adjusted analysis, compared with intending to vote Conservative, being undecided (aOR1.22 [1.13-1.33] <0.001), intending to vote Labour (aOR1.27 [1.16-1.36] <0.001), “Other” (aOR1.54 [1.37-1.72] <0.001), or intending not to vote (aOR1.93 [1.77-2.11] <0.001) was associated with higher odds of smoking relative to never smoking (Table 2). Once more, intending to vote Liberal Democrat or refusing to answer was associated with lower odds of current smoking. Compared with intending to vote Conservative, intending to vote Labour (aOR1.10 (1.03-1.18) =0.006), intending note to vote (aOR1.13 (1.03-1.24) =0.007) and intending to vote ‘Other’ (aOR1.34 (1.21-1.48) <0.001) was each associated with a higher odds of former smoker status.

The results of the adjusted model including all pre-specified covariates revealed a change in direction of odds ratio (OR) for associations between former compared with never smoking and intention to vote Labour, intending not to vote and undecided compared with Conservative, on running further exploratory analyses, this did not appear to be an artefact (see Supplementary Tables 1 and 2).

Furthermore, in the adjusted model compared with intending to vote Conservative, those intending to vote differently were likely to be younger, female (except intending not to vote), in social grades lower than AB, have a lower AUDIT score (except Liberal Democrat voters), and to reside in London.

### Sensitivity analysis

**Fig. 2:**
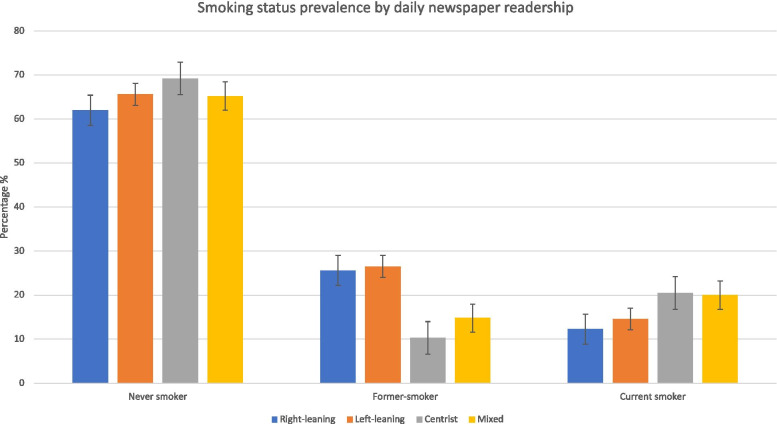
Smoking prevalence (never smoker, former-smoker and current smoker) by daily newspaper readership. Responses were categorised into right-left leaning, centrist or mixed. Bars represent the 95% population proportion confidence interval

In the subgroup of 24,047 respondents selecting one of the newspapers whose political orientation could be classified, the weighted prevalence of reporting reading right-leaning newspapers was 37.5% (95% CI 36.9-38.2), reported left-leaning 33% (95% CI 32.5-33.7), centrist 21.3% (95% CI 20.8-21.8) and mixed readership 8.1% (95% CI 7.8-8.5). Figure 2 shows both centrist and mixed readers had the highest prevalence of current smokers, with 20.5% (95% CI 19.51-21.51) and 20% (95% CI 18.54 - 21.75) respectively. Right-leaning readers had the fewest current smokers 12.3% (95% CI 11.69-12.2).

In fully adjusted model, compared with readers of right-leaning newspapers, left-leaning readers showed no difference in smoking status (Table 3). However, they were significantly more likely to have higher AUDIT scores, be of younger age, male and living in London and to report a social grade lower than AB (except E). Centrist readers showed no difference in current smoking status or AUDIT scores compared with right-leaning readers but had significantly lower odds of being a former-smoker and higher odds of living in London, be male, younger and to report a higher social grade (AB). Mixed readers had significantly higher odds of being a current smoker (no difference in former-smoking status), and also had a higher AUDIT score, be younger and male, living in London and less likely to be in occupation grade lower than AB (except grade E).

**Table 3:** Multinomial regression unadjusted and full adjusted model; newspaper readership, sociodemographic characteristics and alcohol use

	Left leaning		Centrist		Mixed	
	OR (95% CI) *p* value	Adj OR (95% CI) *p* value	OR (95% CI) *p* value	Adj OR (95% CI) *p* value	OR (95% CI) *p* value	Adj OR (95% CI) *p* value
**Smoking status**						
Never smoker	1.00	1.00	1.00	1.00	1.00	1.00
Former smoker	0.83 (0.78-0.89) **<0.001**	1.00 (0.92-1.07) 0.81	0.36 (0.33-0.30) **<0.001**	0.74 (0.65-0.83) **<0.001**	0.55 (0.49-0.62) **<0.001**	0.94 (0.81-1.09) 0.41
Current smoker	1.61 (1.07-1.26) **<0.001**	0.94 (0.85-1.04) 0.23	1.50 (1.38-1.63) **<0.001**	1.09 (0.97-1.22) 0.13	1.56 (1.39-1.76) **<0.001**	1.18 (1.02-1.36) **0.02**
**Age**						
65 +	1.00	1.00	1.00	1.00	1.00	1.00
55-64	1.63 (1.50-1.76) **<0.001**	1.50 (1.37-1.64) **<0.001**	3.82 (3.34-4.36) **<0.001**	3.52 (3.02-4.10) **<0.001**	2.95 (2.49-3.49) **<0.001**	2.53 (2.09-3.05) **<0.001**
45-54	2.47 (2.27-2.69) **<0.001**	2.11 (1.91-2.33) **<0.001**	10.78 (9.51-12.22) **<0.001**	8.44 (7.29-9.77) **<0.001**	5.63 (4.78-6.64) **<0.001**	4.46 (3.71-5.36) **<0.001**
35-44	3.67 (3.31-4.06) **<0.001**	3.06 (2.72-3.43) **<0.001**	24.31 (21.29-27.75) **<0.001**	16.00 (13.70-18.68) **<0.001**	12.21 (10.33-14.42) **<0.001**	8.79 (7.28-10.62) **<0.001**
25-34	3.41 (3.07-3.78) **<0.001**	2.68 (2.38-3.02) **<0.001**	29.99 (26.29-34.22) **<0.001**	15.59 )13.35-18.19) **<0.001**	15.07 (12.79-17.74) **<0.001**	9.48 (7.85-11.43) **<0.001**
16-24	3.03 (2.71-3.40) **<0.001**	2.58 (2.27-2.94) **<0.001**	28.66 (24.99-32.88) **<0.001**	17.69 (15.03-20.81) **<0.001**	16.02 (13.54-18.96) **<0.001**	11.83 (9.74-14.38) **<0.001**
**Sex**						
Female	1.00	1.00	1.00	1.00	1.00	1.00
Male	1.25 (1.18-1.32) **<0.001**	1.14 (1.07-1.22) **<0.001**	1.67 (1.57-1.78) **<0.001**	1.58 (1.46-1.72) **<0.001**	1.22 (1.12-1.34) **<0.001**	1.12 (1.01-1.24) **0.04**
**Occupation social grade**						
AB	1.00	1.00	1.00	1.00	1.00	1.00
C1	0.71 (0.66-0.76) **<0.001**	0.71 (0.65-0.77) **<0.001**	1.44 (1.32-1.57) **<0.001**	1.25 (1.12-1.39) **<0.001**	0.99 (0.88-1.10) 0.79	0.84 (0.73-0.95) **0.007**
C2	0.59 (0.54-0.63) **<0.001**	0.60 (0.55-0.66) **<0.001**	1.57 (1.43-1.72) **<0.001**	1.47 (1.31-1.66) **<0.001**	0.74 (0.65-0.84) **<0.001**	0.73 (0.63-0.85) **<0.001**
D	0.67 (0.61-0.74) **<0.001**	0.70 (0.63-0.78) **<0.001**	2.41 (2.18-2.67) **<0.001**	2.16 (1.89-2.48) **<0.001**	0.73 (0.62-0.86) **<0.001**	0.69 (0.57-0.84) **<0.001**
E	0.80 (0.71-0.90) **<0.001**	0.95 (0.84-1.09) 0.49	1.41 (1.23-1.61) **<0.001**	1.94 (1.62-2.32) **<0.001**	0.83 (0.68-1.01) 0.06	1.06 (0.84-1.33) 0.63
**Region**						
North	1.00	1.00	1.00	1.00	1.00	1.00
Central	0.68 (0.63-0.73) **<0.001**	0.77 (0.70-0.84) **<0.001**	0.73 (0.66-0.82) **<0.001**	0.92 (0.81-1.05) 0.20	0.66 (0.58-0.76) **<0.001**	0.80 (0.68-0.94) **0.005**
South	0.65 (0.61-71) **<0.001**	0.74 (0.68-0.80) **<0.001**	0.69 (0.62-0.77) **<0.001**	1.01 (0.89-1.15) 0.85	0.64 (0.56-0.74) **<0.001**	0.87 (0.75-1.02) 0.80
London	2.26 (2.03-2.44) **<0.001**	2.16 (1.95-2.40) **<0.001**	10.25 (9.27-11.34) **<0.001**	8.02 (7.08-9.08) **<0.001**	5.06 (4.46-5.75) **<0.001**	4.20 (3.61-4.89) **<0.001**
**AUDIT Score**						
	1.06 (1.05-1.06) **<0.001**	1.04 (1.03-1.05) **<0.001**	1.00 (0.98-1.00) **<0.001**	1.00 (0.99-1.01) 0.57	1.05 (1.04-1.06) **<0.001**	1.03 (1.02-1.04) **<0.001**

Right-leaning is the reference category. Bold indicates statistical significance

## Discussion

Relative to those intending to vote Conservative, those intending to vote Labour, for a non-mainstream party and to not vote and those who were undecided were more likely to be smokers and those intending to vote Liberal Democrat were less likely to be smokers, after adjusting for sociodemographic characteristics and alcohol use. Those intending to vote Labour and to not vote, and those intending to vote for an ‘Other’ party, were also more likely to be former smokers compared with those intending to vote Conservative, after adjustment. In relation to newspaper readership, only mixed readership (defined as reading both left and right leaning newspapers) was associated with higher current smoking prevalence. While separately smoking and voting and smoking and newspaper readership show clear associations between smoking and political leanings, when taken together the results are mixed and smoking status cannot be clearly aligned with left, centrist or right leaning political views as a function of both voting intentions and readership.

Our results support previous research in several ways. Evidence from a study in England covering elections across three decades, as well as studies from Ireland, US and Sweden, all demonstrate a strong relationship with intending not to vote and smoking [1, 3, 18, 32]. In our study, 27.4% of those who intended not to vote reported being a current smoker and a low former-smoking prevalence rate (13.8%). Moreover, these current smoking prevalence rates are in contrast to the average smoking prevalence rate in England of 16.5% over the same time period [14], highlighting in England at least, the association between not voting and smoking has remained strong. Current smoking prevalence estimates were also higher in those intending on voting for other parties or Labour (historically the Conservative’s main opposition), and also those who were undecided (19.5%, 17.8%, and 16.3% respectively) compared with intending to vote Conservative. These represent key groups who are at a higher risk of tobacco related morbidity and mortality.

There are several implications from this study; by not voting smokers risk a ‘political exclusion inequality’, which may result in their health and social care needs not being met or prioritised as they lack political representation. This may create a vicious cycle in that by not voting, smokers may feel underrepresented and therefore feel less inclined to vote. For life long smokers, there is also a potential of being less able to vote in older life because of the physical barriers presented by tobacco-related diseases and due to premature death, smokers will also be underrepresented in this demographic [33]. Furthermore, smokers are most represented in those working in occupational grades with lower incomes [13] and who experience structural disadvantages. As well as feeling left behind, as highlighted in the introduction, all parties need to work hard to gain the trust of smokers who may already feel that their tobacco purchasing is mutually beneficial for the government.

The current study provides a useful update to the literature but there are some limitations. Our question was hypothetical (‘If there was an election tomorrow…’) and we did not assess how and if people voted, nor did we adjust for psephologically relevant variables to accurately predict actual voting behaviour. Indeed, 27.1% of our sample suggested they would for Labour but for the duration of the study period Conservatives were the party leading the country, and as such this could reflect the limitations of such a theoretical question or sampling bias. Further, our data were collected over what can be described as a turbulent political period with the Scottish independence referendum, the UK voting to leave the European Union and changes of leadership in several main parties. Consequently, people may have taken different viewpoints than they would have outside of this context. Another limitation is that while we show smoking was related to voting intentions and speculated this was linked to unhealthy behaviours, alcohol was not similarly associated (i.e AUDIT scores were higher among people intending to vote for the Liberal Democrat). This may be an example of the ‘alcohol harm paradox’ [34, 35]. It is commonly demonstrated in the UK that while hazardous drinking is more prevalent among more advantaged occupational grades, harm resulting from drinking is not, these social gradient effects on substance use and socio-political orientation warrant further unpacking. Lastly, the data here derive from a cross sectional survey and therefore do not follow temporal changes.

Future research can expand on these findings by including a wider range of health behaviours, including a more detailed analysis of alcohol use. We included newspaper readership as a sensitivity analysis only, but given the clear role newspapers have in political orientation this deserves more attention than could be given in this paper. For those particularly interested in political orientation and inclusion, the DAG is a useful visual of the environmental stressors individuals and communities face which may preclude them from considering voting for mainstream parties or from participating in elections.

In conclusion, controlling for a range of other factors, smokers as compared with never-smokers appear most likely to intend not to vote, to be undecided, to vote for Labour or a non-mainstream party, and appear least likely to vote for the Conservative Party or the Liberal Democrats. This relationship appears to be predicated on the basis of sociodemographic which separate smokers from those who vote for these two parties.

## Supplementary Information

**Additional file 1.**

## Data Availability

The datasets used and/or analysed during the current study available from the corresponding author on reasonable request.

## Abbreviation

DAG: Directed acyclic graph
