# Supplementary material for: Association between cigarette smoking status and voting intentions: Cross sectional surveys in England 2015-2020
Source: BMC Public Health. 2021 Dec 11;21:2254. doi: 10.1186/s12889-021-12304-4 (PMC8665625; doi:10.1186/s12889-021-12304-4)
Supplement: Supplementary file 1 — Additional file 1. [file 12889_2021_12304_MOESM1_ESM.docx]

**Supplementary file only**

The DAG below represents our assumptions about the causal relations between variables, including the primary ‘exposure’ variable (smoking), outcome (‘voting intention’) and hypothesised confounding and mediating variables. The figure includes observed variables from the present study and also includes those which are deemed important in the causal context of smoking and voting intention but remain unmeasured (latent). The DAG was developed based on evidence from the existing literature and consensus within the research team.

It includes all of the variables that were hypothesised to be key confounders and which, therefore, should be adjusted for future works (family structure, health, risky behaviour, other substance use and voting turnout). Note that the ‘risky behaviour’ variable is latent and indicates the clustering of alcohol and smoking to other health behaviours and may have a downstream impact on voting intentions and participation. Risky behaviours may include, unhealthy diet, life stressors and exposure to adverse events. We include other substance use as a common risk factor within its own right, because use of substances (e.g., cannabis, heroin (27)) is strongly linked to smoking uptake and negatively correlated with cessation. Family structure is also a potential source of residual confounding - smoking is commonly intergenerational (10), as are shared political beliefs and activity (1,4,5). We were unable to adjust for all of these, but they may represent sources of residual confounding.

**Supplementary figure 1: Directed acyclic graph (DAG) depicting which covariates should be included in a multivariable statistical model in order to minimise bias in the estimate of the total casual effect of smoking status on voting intentions. Hypothesised causal pathways are depicted by green lines. Biasing pathways are indicating by pink lines. Exposure (smoking) is in green, outcome (voting intent) is in blue. Observed factors are in white and unobserved latent factors are shown in grey.**


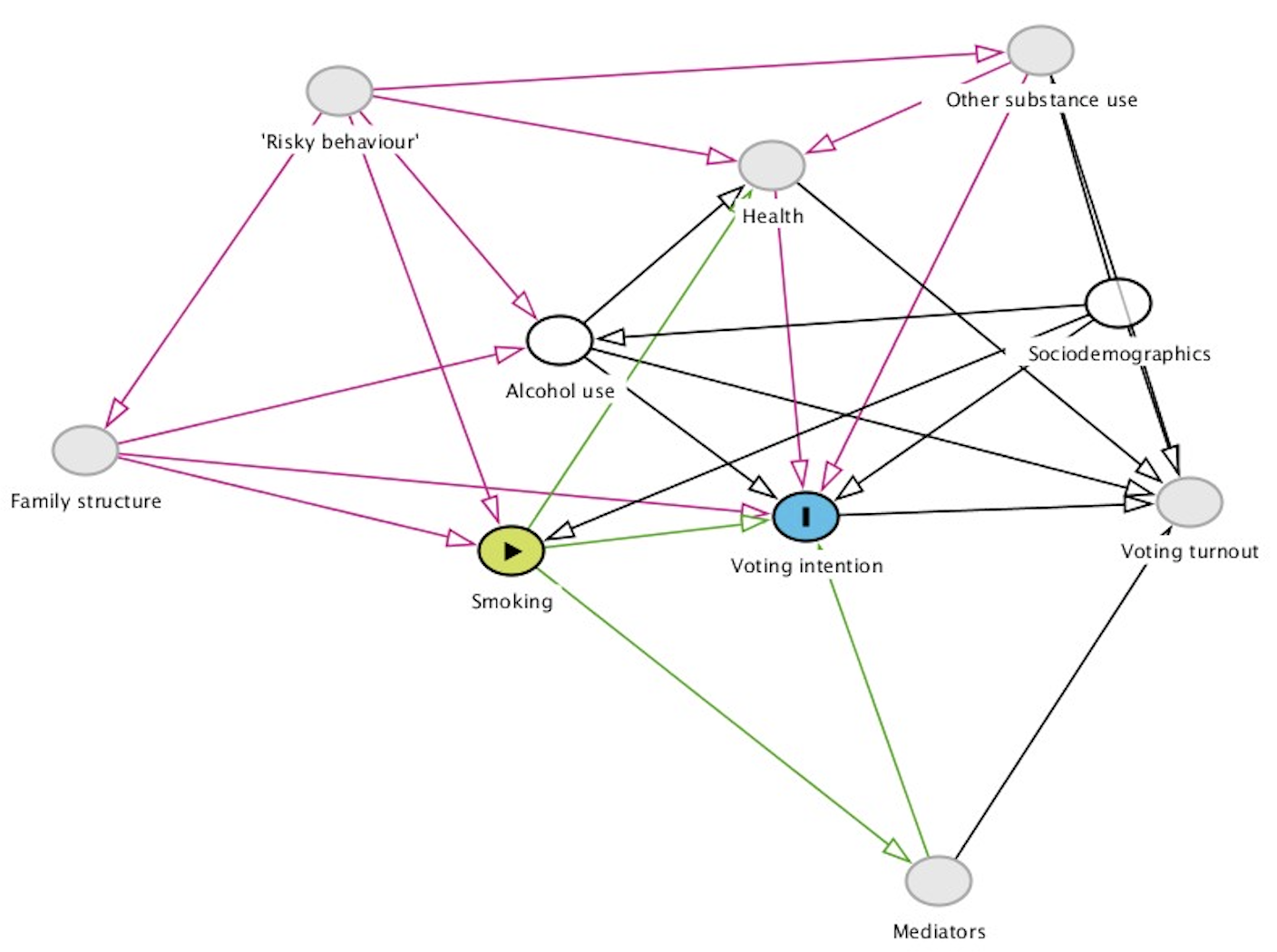


**Supplementary file**

Upon conducing several sensitivity analyses to check model specification. Switching appeared to occur when Age was added as a covariate. The change remained when other covariates were excluded from the model (see supplementary table 1), while the association between age and voting intention in unadjusted analyses remained following adjustment for smoking status (see supplementary Table 2). Both analyses suggest stability in the results. There did not appear to be evidence of over-parameterisation or any issues with multicollinearity. All Generalised VIFs (GVIF; corrected for degrees of freedom) were <2 (GVIF for age 1.7 and for smoking status 1.5), indicating little or no correlation between predictors. Inclusion of age in the adjusted model significantly improved model fit compared to an adjusted model without age (AIC 155751.7 versus AIC 152382.4, i.e. delta or difference >2). Similar results were found when age was entered as a continuous variable and transformations applied (i.e. cubic, quadratic, logarithmic, power and exponential) to ensure a linear relationship with the logit transformation of the outcome.

**Supplementary Table 1:** Multinomial logistic regression results for the association between former-smoking versus never smoking and intention to vote labour with adjustment for age and variations in other confounders.

|  | Labour versus conservative | | | | Intending not to vote versus conservative | | | | Undecided versus conservative | | | |
| --- | --- | --- | --- | --- | --- | --- | --- | --- | --- | --- | --- | --- |
| Former-smoker compared to never smoker | OR | 95%CI Lower | 95%CI upper | P | OR | 95%CI Lower | 95%CI upper | P | OR | 95%CI Lower | 95%CI upper | P |
| Adjusted for:  Age  Gender  Occupation social grade  AUDIT | 1.10 | 0.02 | 1.17 | 0.007 | 1.17 | 1.07 | 1.28 | 0.001 | 1.04 | 0.96 | 1.11 | 0.371 |
| Adjusted for:  Age  Government office region  Occupation social grade  AUDIT | 1.11 | 1.03 | 1.19 | 0.003 | 1.17 | 1.07 | 1.28 | 0.001 | 1.02 | 0.94 | 1.01 | 0.623 |
| Adjusted for:  Age  Government office region  Gender  AUDIT | 1.13 | 1.06 | 1.2 | <0.001 | 1.23 | 1.12 | 1.35 | <0.001 | 1.04 | 0.97 | 1.12 | 0.235 |
| Adjusted for:  Age  Government office region  Gender  Occupation social grade | 1.06 | 0.99 | 1.13 | 0.116 | 1.04 | 0.96 | 1.15 | 0.316 | 0.96 | 0.90 | 1.03 | 0.264 |

**Supplementary Table 2:** Multinomial logistic regression results for the association between age and intention to vote labour with and without adjustment for former-smoking versus never smoking

|  | Labour versus conservative | | | | Intending not to vote versus conservative | | | | Undecided versus conservative | | | |
| --- | --- | --- | --- | --- | --- | --- | --- | --- | --- | --- | --- | --- |
| Age | OR | 95%CI Lower | 95%CI upper | p | OR | 95%CI Lower | 95%CI upper | P | OR | 95%CI Lower | 95%CI upper | P |
| Adjusted for smoking status  65+  16-24  25-34  35-44  44-54  55-64 | 1  7.09  5.77  4.00  2.43  1.74 | 6.40  5.21  3.64  2.23  1.61 | 7.85  6.28  4.39  2.65  1.89 | <0.001  <0.001  <0.001  <0.001  <0.001 | 1  9.41  8.04  4.57  2.52  1.77 | 8.29  7.09  4.04  2.23  1.57 | 10.69  9.11  5.18  2.85  1.99 | <0.001  <0.001  <0.001  <0.001  <0.001 | 1  4.24  4.03  2.86  1.90  1.50 | 3.82  3.62  2.60  1.74  1.38 | 4.71  4.48  3.14  2.07  1.63 | <0.001  <0.001  <0.001  <0.001  <0.001 |
| Not adjusted for smoking status  65+  16-24  25-34  35-44  44-54  55-64 | 1  7.05  5.75  3.99  2.42  1.74 | 6.37  5.20  3.63  2.22  1.60 | 7.80  6.36  4.37  2.64  1.89 | <0.001  <0.001  <0.001  <0.001  <0.001 | 1  9.34  7.99  4.56  2.52  1.76 | 8.24  7.06  4.03  2.23  1.56 | 10.57  9.05  5.15  2.84  1.99 | <0.001  <0.001  <0.001  <0.001  <0.001 | 1  4.32  4.09  2.89  1.91  1.51 | 3.89  3.69  2.62  1.76  1.39 | 4.79  4.53  3.17  2.09  1.63 | <0.001  <0.001  <0.001  <0.001  <0.001 |
